# Delayed adverse events in phase I trials of molecularly targeted and cytotoxic agents

**DOI:** 10.18632/oncotarget.26104

**Published:** 2018-09-21

**Authors:** Emma J. Welsh, James Spicer, Debashis Sarker

**Affiliations:** ^1^ School of Cancer and Pharmaceutical Sciences, King's College London, London, UK; ^2^ Guy's and St Thomas' NHS Foundation Trust, Guy's Hospital, London, UK

**Keywords:** adverse events, phase I trials, molecularly targeted agents, cytotoxic agents

## Abstract

**Background:**

Grade 3 and 4 adverse events (AEs) during cycle 1 are traditionally used for dose escalation decisions in Phase I oncology trials. With molecularly targeted agents (MTAs), assessment of lower grade AEs and those in later cycles is considered increasingly relevant.

**Methods:**

We conducted a retrospective analysis of AEs in patients enrolled onto relevant phase I trials of MTAs and cytotoxic combinations (CCs) at our UK centre between 2006 and 2016. All AEs in the first six cycles deemed at least ‘possibly related’ were recorded.

**Results:**

A total of 912 AEs were identified in 127 patients across 15 trials. Mean AE totals for CCs or MTAs respectively was 4.7 versus 3.0 in cycle 1, 3.8 versus 2.8 in cycles 2-6. Patients on CCs had higher mean AEs in six cycles compared to those on MTAs (8.5 vs. 5.7, p = 0.0005). For patients experiencing grade 3 AEs, 58% (CCs) and 60% (MTAs) occurred for the first time after cycle 1.

**Conclusion:**

Overall AE incidence was lower in MTAs than CCs across six cycles. For MTAs, more frequent incidence of first grade 3/4 AEs after cycle 1 supports incorporation of delayed AEs into recommendations for Phase 2 dosing.

## INTRODUCTION

Adverse events (AEs) in early phase oncology trials play a key role in dose escalation [1], with dose-limiting toxicities (DLTs) being particularly important. While AEs and DLTs are recorded throughout the entirety of a trial, occurrences beyond the DLT window (cycle 1) are rarely incorporated into subsequent publication [2].

It has been established that there is variation in DLTs between cytotoxic and non-cytotoxic agents [3]. Cytotoxic agents generally exhibit more severe and higher grades of toxicity in earlier cycles [4], whereas molecularly targeted agents (MTAs) are often administered chronically until progression, making consideration of lower grade AEs and those in later cycles highly relevant [2, 5, 6]. This has led to investigators having to consider how to address both late and dose-limiting toxicities across drug types [7]. Some investigators have suggested the classic DLT window should be extended to take into account MTAs toxicity [2, 8]. However, differences in the timing and severity of cytotoxic and MTAs AEs suggest a need for flexibility in managing different trial types [9].

To our knowledge, there has been no previous investigation into differences in AEs in phase I trials of MTAs compare to cytotoxic combinations (CCs) over time. The primary objective of this study was to analyse AEs from patients enrolled on relevant phase I dose escalation trials at a single UK centre to determine incidence of AEs between drug classes (CCs versus MTAs), and between cycle 1 versus cycles 2-6.

Previous work has compared single agent cytotoxic and MTA dermatological AEs and found a substantial number of AEs occurring outside the DLT window with variation in AE type based on agent studied [10]. It has been suggested that toxicities from all cycles should be taken into account for analysis and reporting of phase I trials [11].

In line with previous work suggesting ethnic, age and gender based risk of toxicities [12], the secondary objective of this study was to determine differences in incidence of AEs across specific clinical, demographic and socio-economic cohorts.

## RESULTS

### Patient demographics

**Figure 1:**
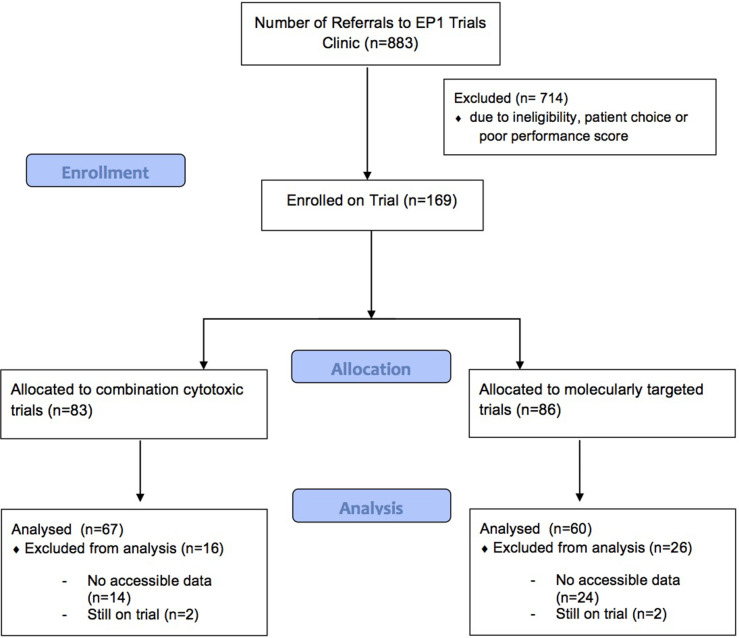
CONSORT flow diagram Flow of patients selected for analysis in this study.

A total of 127 patients' records were accessed; of these, 67 patients were enrolled onto CCs and 60 patients were enrolled onto MTAs trials (Figure 1). Patients on CCs completed a total of 241 cycles and patients on MTAs completed a total of 179 cycles. Average cycles completed was 4.0 in CCs and 3.0 in MTAs. Of 127 patients, 52 (40.9%) were men and 75 (59.0%) were women, with a mean average age at trial start of 59 years (61.1 years for men and 57.6 years for women). Table 1 shows other baseline characteristics relevant to this study.

**Table 1:** Patient demographics

	N (%)
**Age**	
25-34	4 (3.15)
35-44	14 (11.02)
45-54	21 (16.54)
55-64	46 (36.22)
65-74	30 (23.62)
75-84	12 (9.45)
**Gender**	
Male	52 (40.94)
Female	75 (59.06)
**Ethnicity**	
Caucasian	91 (71.65)
Not Caucasian	15 (11.81)
Not Specified	21 (16.54)
**Charlson Comorbidity Index Score**	
0	85 (66.93)
1	21 (16.54)
≥2	3 (2.36)
Unknown	18 (14.17)
**Index of Multiple Deprivation Score**	
1 (least deprived)	22 (17.32)
2	22 (17.32)
3	21 (16.54)
4	36 (28.35)
5 (most deprived)	21 (16.54)
Not Stated^a^	5 (3.93)
**Number of Metastatic Sites**	
0	1 (0.79)
1	39 (30.71)
2	46 (36.22)
3	23 (18.11)
4	5 (3.94)
5	1 (0.79)
Unknown	12 (9.44)
**Previous Radiotherapy**	
Yes	39 (30.71)
No	70 (55.12)
Unknown	18 (14.17)
**Previous Lines of Chemotherapy**	
1	39 (30.71)
2	30 (23.62)
3	18 (14.17)
4	12 (9.45)
≥5	11 (8.66)
Unknown	17 (13.39)
**Neutrophil-Lymphocyte Ratio**	
<5	75 (59.06)
≥5	33 (25.98)
Unknown	19 (14.96)
**Royal Marsden Hospital Score**	
Low	90 (70.87)
High	19 (14.96)
Unknown	18 (14.17)

Demographics of the 127 patients referred to the early phase trials unit at Guy's Hospital London who subsequently went on a dose escalation trial and had their adverse events recorded for this study.

^a^ – includes patients who did not have an English postcode.

### Trial characteristics and cycles completed

Patients were enrolled on a total of 15 trials (7 CCs and 8 MTAs). Details of specific MTAs pathways and cytotoxic drug mechanisms of action can be found in Table 2.

**Table 2:** Trial characteristics

	Patients (%)/Trials
**Cytotoxic Combination Trials (N=7)**	
**Cytotoxic Agents**	
Platinum + Taxane	28 (41.8) / 1
Cyclophosphamide	19 (28.4) / 3
Taxane	20 (33.3) / 3
**Molecularly Targeted Agent Trials (N=8)**	
**MTA Pathways**	
HER Inhibitor	27 (45.0) / 2
Notch Inhibitor	4 (6.7) / 1
ATR Inhibitor	2 (3.3) / 1
PI3 Kinase Inhibitor	3 (5.0) / 1
PARP Inhibitor	13 (21.6) / 1
CYP17A1 Inhibitor	9 (15.0) / 1
FGFR4 Inhibitor	2 (3.3) / 1

Characteristics of trials in which the 127 patients analysed in this study were enrolled onto and participated in, with specific divisions the type of cytotoxic agent used and the pathways of the molecularly targeted agents.

Of 67 patients who begun a CC trial, all completed cycle 1, 67 (83.6%) progressed to cycle 2, 39 (58.2%) progressed to cycle 3, 32 (47.8%) to cycle 4, 26 (38.8%) to cycle 5 and 21 (31.3%) completed all six cycles.

In the MTAs trials, all 60 patients completed cycle 1 successfully, with 52 (86.6%) progressing to cycle 2, 27 (45.0%) reaching cycle 3, 21 (35.0%) completed cycle 4, 11 (18.3%) in cycle 5 and a total of eight (13.3%) patients completed all six cycles.

Failure to complete six cycles of treatment was due to either progressive disease or toxicity.

### Adverse events

A total of 912 AEs recorded in 127 patients. By trial, 568 AEs were recorded from the 67 patients on CCs, 316 (56.6%) of these occurred during cycle 1, while 252 (44.4%) occurred within cycles 2-6. A total of 344 AEs were recorded in 60 patients on MTAs, 177 (51.5%) occurred within cycle 1 and 167 (48.5%) in cycles 2-6.

The highest AE total in an individual patient on CCs was 20 (range 0-20), 16 for a patient on MTAs (range 0-16). Mean average AE total per patient on CCs for cycle 1 and cycles 2-6 were 4.7 and 3.8 respectively, while average AE total per patient on MTAs trials in cycle 1 was 3.0, with a 2.8 average in cycles 2-6.

Average AE totals for six cycles were 8.5 and 5.7 for patients on CCs and MTAs trials respectively (p = 0.0005), indicating that patients on CCs experienced a significantly larger number of AEs in six cycles compared with those on MTAs trials.

**Figure 2:**
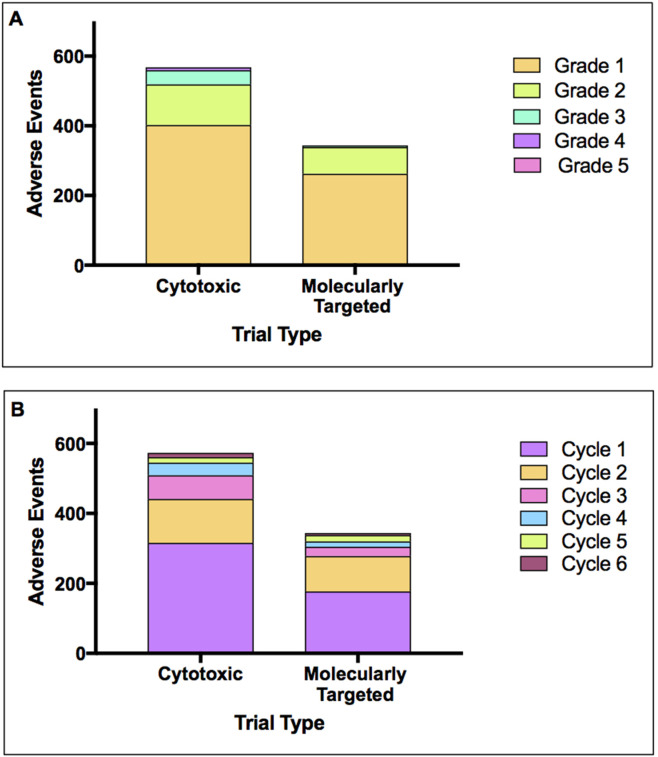
Adverse events by trial type Adverse events collected from patients of each trial type, cytotoxic combination and MTAs single agent, split by **(A)** CTCAE grade 1-6 and **(B)** cycle number 1-6.

Figure 2A shows AEs split by trial type, either CCs or MTAs. AEs recorded were split by CTCAE grade with grades 1 and 2 being most prevalent in both groups. AEs were then split by cycle number (Figure 2B) and arranged by trial type, either CCs or MTAs. AEs in cycles 1 and 2 accounted for the majority with AEs occurring throughout all cycles in both groups.

Table 3 shows AEs ranked by incidence in cycle 1 and cycles 2-6. Fatigue and diarrhoea showed high incidence in all cycles. Beyond cycle 1, AEs such as anorexia and vomiting became more apparent in all patients.

**Table 3:** Top 10 adverse events

Cytotoxic Combination Trials (N=67)				Molecularly Targeted Single Agent Trials (N=60)			
Cycle 1	n (%)	Cycles 2-6	n (%)	Cycle 1	n (%)	Cycles 2-6	n (%)
Diarrhoea	36 (53.7)	Fatigue	24 (35.8)	Fatigue	25 (41.7)	Fatigue	20 (33.3)
Rash^a^	32 (47.8)	Diarrhoea	19 (28.4)	Diarrhoea	19 (31.7)	Anorexia	12 (20.0)
Fatigue	28 (41.8)	Mucositis^b^	18 (26.9)	Nausea	18 (30.0)	Diarrhoea	10 (16.7)
Mucositis^b^	23 (34.3)	Alopecia	14 (20.9)	Rash^a^	14 (23.3)	Rash^a^	9 (15.0)
Nausea	22 (32.8)	Rash^a^	12 (17.9)	Mucositis^b^	9 (15.0)	Vomiting	9 (15.0)
Pyrexia	16 (23.9)	Vomiting	12 (17.9)	Anorexia	8 (13.3)	Nausea	9 (15.0)
Anorexia	11 (16.4)	Nausea	10 (14.9)	Dysgeusia	6 (10.0)	Oedema	6 (10.0)
Constipation	9 (13.4)	Anorexia	10 (14.9)	Urine Discoloration	5 (8.3)	Increased Bilirubin	5 (8.3)
Epistaxis	8 (11.9)	Anaemia	9 (13.4)	Dry Skin	5 (8.3)	Mucositis^b^	5 (8.3)
Neutropenia^c^	8 (11.9)	Neutropenia^c^	8 (11.9)	Dyspepsia	5 (8.3)	Pain	5 (8.3)

Ranked adverse events by trial type and cycle number, either cycle 1 or cycles 2-6. AEs were ranked to show top 10 in each trial and cycle group. AEs are displayed in ascending order of raw count with percentage incidence given in brackets.

^a^ including pruritic, fungal, macropapular, acneiform, pustular.

^b^ including stomatitis and coryzal symptoms.

^c^ including resultant fever or sepsis.

### Time to first adverse event

**Figure 3:**
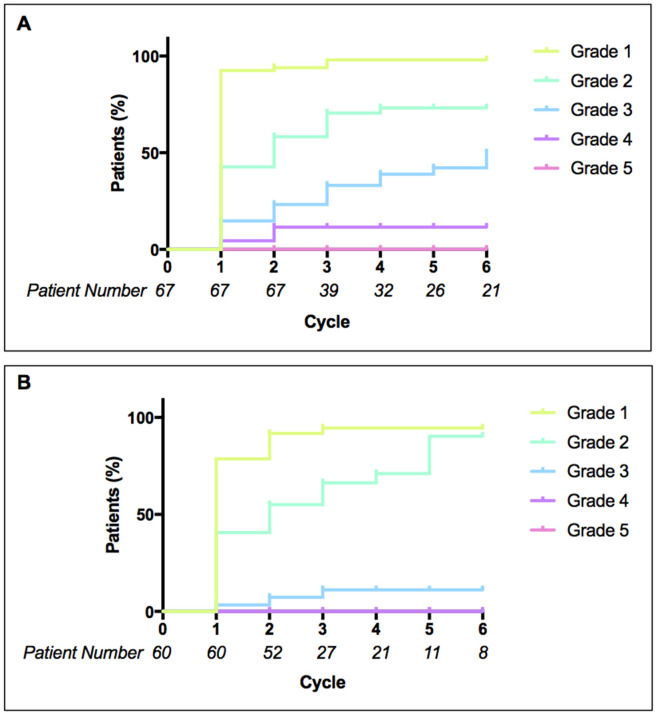
Time to first adverse event Cumulative incidence (in percentage) of patients experiencing their first adverse event of each grade (1-5) in cycles 1 to 6 of **(A)** cytotoxic combination trials and **(B)** molecularly targeted agent trials. Taking into account patients who have already experienced adverse events of each grade and patients who were no longer on treatment at each cycle.

Cumulative AE incidence by grade was evaluated by the creation of time to first AE curves. For CCs (Figure 3A), of the 24 patients who experienced G3 toxicity, 58% presented for the first time after cycle 1. Incidence of G4 toxicity beyond cycle 1 was of a similar occurrence, with 43.0% of patients with G4 toxicity presenting for the first time in cycles 2-6.

In MTAs trials (Figure 3B), 34.0% of patients who experienced G2 toxicity presented following cycle 1 (taking into account decreasing number of patients per cycle); there was a gradual increase of first incidence G2 toxicity throughout all six cycles. G3 toxicity incidence was low overall (5 patients) but three events occurred for the first time beyond cycle 1. Log-rank (Mantel-Cox) tests were performed to compare incidence of each grade between trial types; G1, G2 and G5 all showed no significant difference (p-values > 0.05). G3 showed a highly significant difference (p-value 0.0033) between CCs and MTAs trials; G4 toxicity also showed a significant difference between CCs and MTAs (p-value 0.01).

### Adverse events across clinical, demographic and socioeconomic variables

Statistical analyses were performed across a range of clinical, demographic and socioeconomic variables (Table 1) comparing mean AE values in cycle 1, cycles 2-6 and overall. There were no statistically significant differences (p-values > 0.05) in AEs across age, gender, ethnicity, comorbidity index, depravation index, number of metastatic sites, prior radiotherapy or chemotherapy, NLR and RMH scores.

### Treatment interruptions and dose modifications

**Figure 4:**
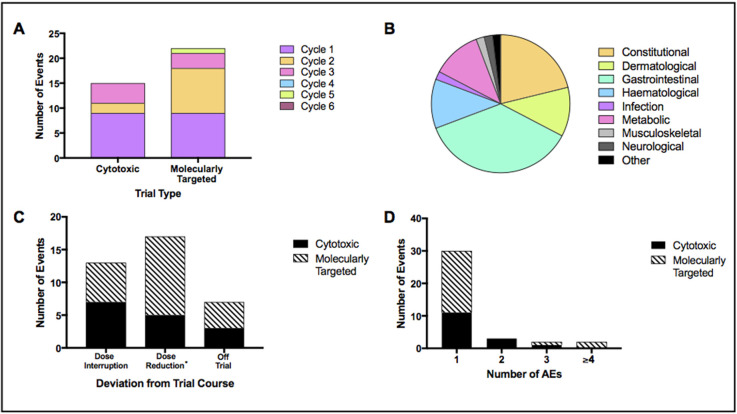
Treatment interruptions and dose modifications Display of several variables relating to treatment interruptions and dose modifications. Events included dose interruptions, dose reductions or removal of patient from trial. Results displayed as; **(A)** events by trial type – cytotoxic combination or molecularly targeted single agent; **(B)** AEs contributing to all events split by category; **(C)** events by type – dose interruption, dose reduction (^*^including dose interruptions that resulted in dose reductions) and removal of patient from trial; **(D)** events by number of contributing AEs.

Events that were directly caused by toxicity, resulting in dose interruptions, dose reductions or a removal of a patient from the trial they were enrolled in, were recorded. These interruptions and modifications consisted of 52 AEs, resulting in 37 events in a total of 28 patients. AEs involved in these events were split by grade; G1 = 5, G2 = 26, G3 = 20, G4 = 1 and G5 = 0. These AEs spanned nine different categories (Figure 4B), with constitutional and dermatological events accounting for the majority (57.7%).

Figure 4A shows events split by trial type. Of 37 events, 41.0% occurred in patients on CCs and 59.0% in patients on MTAs trials. Events were spread throughout cycle 1, 2 and 3 in patients on CCs whereas events were also recorded in cycle 5 for patients in MTAs trials. The majority of events in patients on CCs occurred within cycle 1 (60.0%) however the majority of events in those on MTAs occurred beyond cycle 1, with a total of 13 events (59.0%) in cycles 2-5 (p > 0.05).

Figure 4C shows the distribution of event types by trial (dose interruptions, dose reductions and off trial). Patients on CCs experienced a higher incidence of dose interruptions (46.6% versus 27.3% for patients on MTAs). Dose reductions were the highest in patients on MTAs trials, accounting for 54.5% (12/22 events), and 33.3% (5/15) of events in CCs. Number of AEs contributing to each event is shown in Figure 4D. Highest AE total in one event was six and was recorded in a patient on a MTAs trial.

### Dose limiting toxicities

Of 37 events causing treatment interruptions or dose modifications, 17 were classified as DLTs, 10 occurring within the DLT window (cycle 1) and 7 in cycles 2-6.

Split by trial type, 50% of traditional DLTs occurred on CCs and 50% occurred in those on MTAs trials. Of the DLT events beyond cycle 1, 43.0% were recorded in patients on CCs and the remaining 57.0% in patients on MTAs trials. Irrespective of cycle, DLT events in patients on CCs accounted for 1.4% of all AEs, whereas for patients on MTAs trials DLT events made up 2.6% of all AEs.

## DISCUSSION

Evaluation of the differences of adverse events in cytotoxic and molecularly targeted agents is a prominent consideration in early phase trials. Cytotoxic AEs typically occur more acutely and show higher incidence of increased severity [4], whereas MTAs AEs present more chronically with a lower level of toxicity [13].

Previous studies have evaluated specific aspects of toxicity patterns in phase I trials, such as the emergence of MTAs AEs [14] and variation in DLT events [3]. With that in mind, this study set out to investigate a broader toxicity perspective by including all AEs recorded.

To our knowledge, this is the first study to assess differences in incidence of AEs between MTAs and CCs, and across different cycle numbers. A total of 912 AEs were recorded across 420 cycles of treatment in 127 patients. We have confirmed that patients on CC trials experienced a significantly higher number of AEs over six cycles compared with MTAs. This is in keeping with previous data from other series, supporting findings that patients on CCs experience a higher volume of AEs [15] than MTAs [16]. These findings were supported by a significantly higher cumulative incidence of patients on CC trials experiencing their first G3 and G4 toxicity over six cycles. Incidence of first G2 and G3 toxicities in patients on MTAs trials increased steadily throughout cycles 2-6, highlighting the later presentation of toxicities with MTAs [2].

Treatment interruptions and dose modifications showed no significant difference between trial types or cycles, however the numbers of patients in these subgroups were too small to draw meaningful comparisons. Nevertheless there was still a notable amount of these events following the completion of cycle 1 in both trial types, an important finding given that dose interruptions and reductions can lead to suboptimal therapeutic outcomes [17]. DLT comparisons between both trial types (CCs versus MTAs) and cycles (cycle 1 versus cycles 2-6) support previous work, indicating that the DLT period is not long enough and should be extended beyond the traditional DLT window of cycle 1 [8].

The main limitation of this study is the lack of prospective recording of asymptomatic haematological and biochemical abnormalities. Although this is unlikely to interfere with the conclusions reached, in the future these laboratory results should be prospectively CTCAE graded and included for a broader evaluation of all AEs. In addition, the retrospectively analysis of AEs is another limitation of this study; prospective analysis of both symptomatic and laboratory AEs would allow for more accurate information to be recorded for each patient.

Age and ethnicity have been suggested to influence toxicity [12] in a large variety of drugs not just chemotherapeutic agents [18–20]. In addition, number of metastatic sites, NLR and RMH scores are important indicators of prognosis [21]. These variables were therefore analysed for differences in toxicity. Gender, comorbidity index score, socioeconomic status, radiotherapy history and chemotherapy history were also evaluated. In the cohorts studied, there was no significant difference found between any subgroup of the variables tested, potentially due to small sample size. Although these results are comparatively different to some previous studies [12], this lack of significant difference in toxicity may be beneficial with regards to enrolment of patients on phase 1 trials. Previous work from our centre has demonstrated that patients from a lower socioeconomic background (based on index of multiple depravation) are statistically less likely to be referred to a trials unit [22].

The results of this study provide an evaluation of drug toxicities between patients treated in cytotoxic combination and MTA trials. The data presented here, showing more frequent incidence of first grade 3-4 AEs after cycle 1 for MTAs, supports the routine incorporation of delayed AEs into recommendations of Phase 2 dosing and schedule for these agents.

## MATERIALS AND METHODS

### Patients and trial characteristics

Patients enrolled on phase I dose escalation trials in the early phase oncology trials unit at Guy's Hospital, London, United Kingdom between March 2006 and May 2016 were selected. For patients enrolled onto multiple trials, only the first trial was included for data collection.

127 patients were identified as those who were enrolled onto phase I dose escalation trials, completed at least one cycle of treatment and had accessible source data for recording (Figure 1). Seventy patients were entered onto a total of seven combination trials containing cytotoxic agents and 60 patients were entered onto a total of eight single agent trials involving MTAs.

All patients were anonymised to maintain confidentiality.

### Data collection

All data was collected on a retrospective basis using computer sources including the electronic patient record system (EPR) alongside the oncology and haematology clinical information system (CIS). Paper sources of data included source data files from the local and external Guy's and St Thomas' NHS Foundation Trust (GSTT) trial archives.

#### Adverse events

All AEs that were deemed at least ‘possibly related’ and were not classified as present at baseline were recorded throughout cycles 1 to 6. All AEs were graded using the Cancer Institute Common Terminology Criteria of Adverse Events (CTCAE), version 3.0 (4 trials) or version 4.3 (11 trials).

#### Other variables

Information regarding patient variables such as comorbidity index, neutrophil-lymphocyte ratio (NLR), Royal Marsden Hospital (RMH) score and socioeconomic status was collected. Socioeconomic status was assessed using the Index of Multiple Depravation (IMD) tool, provided online by the University of Oxford National Perinatal Epidemiology Unit (NPEU), which calculates IMD based on English postcodes.

### Data analysis

Raw AE data was organised and manipulated to provide total AE values per patient to a total of up to six cycles. Total number of cycles completed was recorded for each patient and total AEs per cycle calculated.

All results were transferred to the statistical analysis software GraphPad Prism 7 (GraphPad Software, Inc.; California, USA), where data was organised to calculate specific results and statistical analyses were performed where necessary. Multiple unpaired two-tailed t-tests and one-way ANOVA tests were performed to identify any significant difference in AE incidence within the subgroups of the following variables; age, gender, ethnicity, comorbidity index, depravation index, number of metastatic sites, radiotherapy history, chemotherapy history, NLR and RMH scores.

Analysis of time to first AE data was performed using log-rank (Mantel-Cox) tests alongside a log-rank test for trend. For analysis of treatment interruption and dose modification events between trial types, two-way ANOVA and further t-tests were performed.

For all results, a p-value of ≤0.05 was deemed to be significant.

## Abbreviations

AE: adverse events
DLT: dose-limiting toxicities
MTA: molecularly targeted agent
CC: combination cytotoxic
CTCAE: common terminology criteria for adverse events
NLR: neutrophil lymphocyte ratio
RMH: Royal Marsden Hospital Score
EPR: electronic patient record
CIS: clinical information system
GSTT: Guy's and St Thomas' NHS Foundation Trust
